# Current Status of Endothelin Receptor Antagonists in Pulmonary Arterial Hypertension: A Combined Study Results and Pharmacology-Based Review

**DOI:** 10.7759/cureus.42748

**Published:** 2023-07-31

**Authors:** Shamsun Nahar, Srishti Kanda, Uzair Chatha, Victor A Odoma, Aakanksha Pitliya, Esraa M AlEdani, Japneet K Bhangu, Khalid Javed, Prabhleen Kaur Manshahia, Ann Kashmer Yu

**Affiliations:** 1 Internal Medicine, California Institute of Behavioral Neurosciences and Psychology, Fairfield, USA; 2 Medicine, Lahore Medical and Dental College, Lahore, PAK; 3 Cardiology/Oncology, IU Health, Bloomington, USA; 4 Anesthesiology, Internal Medicine, St. George's University School of Medicine, Chicago, USA; 5 Medicine, All India Institute of Medical Sciences, Rishikesh, Rishikesh, IND; 6 Internal Medicine, Jean-Charles Medical Center (JCMI), Orlando, USA

**Keywords:** macitentan, efficacy and safety, ambrisentan, bosentan, endothelin receptor antagonists, treatment of pulmonary hypertension, pulmonary arterial hypertension

## Abstract

Pulmonary arterial hypertension (PAH) affects a wide range of people globally and has a poor prognosis despite many advancements in available treatment options. Among the available treatments, endothelin receptor antagonists (ERA) are among the most widely used drugs. These drugs have been evaluated in scientific trials. We included free full texts in the English language from the last ten years and reviewed them. We are writing this review to amalgamate the pharmacological aspects and the previous studies on ERAs to demonstrate a comprehensive overview of the current status of ERAs for PAH treatment. We focused on the structure, pharmacodynamics, pharmacokinetics, and efficacy and safety of the three most widely used ERAs: Bosentan, Ambrisentan, and Macitentan. These drugs have different receptor affinities, bioavailability, excretion routes, and different levels of safety profiles. There are three available studies, the RCT, the ARIES series, and the SERAPHIN studies, for assessing the safety and efficacy of Bosentan, Ambrisentan, and Macitentan, respectively. All the studies and some additional studies for combination therapy have proven all three drugs effective in treating PAH. The side effects (SE) varied from headache and hepatic enzyme elevation to worsening the PAH status of varied severities. Although these studies provided valuable insight into the role of ERAs, there is still enough scope for more studies on ERAs, both as monotherapy and combination therapy for PAH.

## Introduction and background

Pulmonary arterial hypertension (PAH) has a complex pathophysiology and diverse treatment options. Patients with PAH have a mean life duration of about three years from the diagnosis, as mentioned by Hanna et al. [1]. According to Duo-Ji and Long, approximately 15 people per million suffer from PAH [2]. While Maron et al. noted that the prevalence and incidence of PAH are 25 per million and five cases per million per year, several risk factors such as family, social, and past medical histories can play a pivotal role in PAH development, according to McGoon et al. Ruopp and Cockrill described that PAH is associated with cumulative pulmonary artery remodeling followed by a right ventricular compensatory afterload increase [3-5]. That is evidenced by finding precapillary pulmonary hypertension during a right heart catheterization (RHC), where the mean pulmonary arterial pressure is ⩾25 mmHg at rest, the wedge pressure ⩽15 mmHg, and the pulmonary vascular resistance is >3 Wood units despite the absence of any other cause of precapillary pulmonary hypertension such as chronic thromboembolic pulmonary hypertension or lung disease [6-8].

According to Ruopp and Cockrill [5] and Elahsboury and Anderso [9], the most prevalent presentation of PAH is dyspnea during exertion, followed by dyspnea at rest as the disease progresses [5]. Other symptoms include weakness, fatigue, exercise intolerance, syncope, and anginal chest pain. A comprehensive examination must include a detailed family history, medical history, and physical examination to screen for PAH. The initial diagnostic test is an echocardiogram, and if signs of PAH are present, the echocardiogram can determine if that is due to congenital causes of left heart disease. Then additional tests may be done, such as HIV and antinuclear antibody serology, ventilation/perfusion scans, pulmonary function tests (PFT), liver function tests (LFT), and overnight oximetry, to detect the specific etiology of PAH. The confirmatory test to diagnose and determine the disease severity is right heart catheterization, which also helps to decide the treatment guidelines [9].

The PAH is first among the WHO PAH diagnostic classification groups, as described by Belge and Delcroix [6] and Seferian and Simonneau [10]. Among all the subtypes of PAH, no cause is found for the idiopathic form, while the heritable condition has a backdrop of genetic mutation or family history. Other etiologies include toxins and drugs, liver diseases, connective tissue diseases (CTDs), the human immunodeficiency virus (HIV), or schistosomiasis [5-7].

As per Elahsboury and Anderson, the prognosis and progression of PAH are not linear, and the clinical status and severity are classified according to the World Health Organization's (WHO) functional classification (FC) scale. Progression from class I to class IV leads to increased disease severity. Classification I has the asymptomatic to least restrictive symptoms in activities of daily living. In contrast, classification IV comprises the most functionally impaired PAH patients, who are symptomatic even at rest. Classifications II and III refer to mild and marked functional impairments, respectively. The prognosis is best to worst according to WHO FC, while classes I to IV have the worst prognosis [9].

PAH pathogenesis usually results from an imbalance between the primary mediators of pulmonary arterial circulation, which leads to increased vasoconstriction, thrombosis, and proliferation of endothelial cells and smooth muscle within pulmonary vessels. Endothelin is a mediator that plays a vital role in smooth muscle proliferation. The gradual remodeling of the smaller distal pulmonary vessels (diameter <500 mm) eventually leads to increased pulmonary vascular resistance, followed by right heart failure [7,10,11]. The proliferation of the intima and media, leading to pulmonary vascular obstruction, is a principal mechanism involved in PAH pathogenesis [7]. Endothelin-1 (Et-1) levels are elevated in PAH patients and contribute to the advancement of PAH. There is a proportionate decrease in pulmonary arterial circulation and cardiac output when there is an increase in the level of Endothelin-1 in the pulmonary circulation. So, therapies to treat PAH are recently focusing on blocking the harmful effects of Endothelin-1, as per the opinion of Elahsboury and Anderson [9].

The treatment goals for PAH are to improve symptoms, functional quality of life, survival, slow disease progression, and prevent right heart failure. The treatment response is measured objectively by improvement in WHO FC (which is a mirror of the New York Heart Association [NYHA] functional classes of heart failure), capacity to exercise, six-minute walking distance (6MWD), cardiopulmonary exercise test, and treadmill test, hemodynamics obtained from RHC, and survival. According to McGoon et al., worse prognostic outcomes include >65-year-old males with etiologically CTD, a higher WHO FC or NYHA FC, a more elevated B-type natriuretic peptide (BNP) level and N terminal pro-BNP level (NT pro-BNP), radiologically pericardial effusion on echocardiography, and a lower predicted value. Of the diffusing capacity of the lung for carbon monoxide (DLCO) and some hemodynamic parameters like lower cardiac output (CO) or cardiac index (CI) [3].

Few current drugs are available that decreased the mortality rate, while many showed improved 6MWD. Though this measurement may be variable and questioned, WHO groups 2-5 treatment data are not as readily abundant as in PAH, as no therapies are currently available or approved for treating these PAH classes. At present, there are three drugs available approved by the organization named USA Food and Drug Administration (FDA) approved for chronic PAH treatment as follows: prostacyclin analogs, endothelin receptor antagonists (ERAs), and phosphodiesterase type-5 inhibitors (PDE5 I), according to Duarte et al. [7,11,12].

Currently, approved ERA drugs for treating PAH include Bosentan, Ambrisentan, and Macitentan. Bosentan was the first drug to be available and was associated with a dose-dependent increase in aminotransferases; Macitentan and Ambrisentan were also associated with rare hepatotoxicity, as per Chen et al. [13]. Other joint SEs include increased plasma bile salts and alkaline phosphatases, headaches and flushing, peripheral edema, sinusitis, and nasal congestion, varying in severity ranging from mild to moderate [1,6,9].

There need to be more articles combining a detailed discussion of the pharmacological aspects of ERAs with the results of the available studies on the efficacy and safety of ERAs. We will combine both of these sectors of ERAs, which will be beneficial for future research.

## Review

Method

We used several keywords such as PAH, treatment of pulmonary hypertension, combination therapy of PAH, endothelin receptor antagonists (Bosentan, Ambrisentan, and Macitentan), efficacy and safety of ERAs, hepatotoxicity of ERAs, and epidemiology of PAH to find data. We used these keywords alone or in combination to search data on two databases: PubMed and Google Scholar. After reviewing the articles using the title and abstract, we selected hundreds of records. Later, we reviewed the articles again based on criteria such as the last ten years, the English language, and free full texts. Finally, we selected relevant articles after screening based on the requirements mentioned earlier and reviewed the articles individually based on our study topics. Table 1 illustrates a summary of the selected articles.

**Table 1:** Summary of the included studies RCRPC: respirable controlled release polymeric colloid, ERA: endothelin receptor antagonists, PAH: pulmonary arterial hypertension, 6MWD: six minutes walking distance, NIH: National Institutes of Health, PPH: primary pulmonary hypertension, WHO: World Health Organization, FC: functional class, SE: side effects, PVH: pulmonary vascular hypertension, PDE5: phosphodiesterase 5, NT-pro BNP: N-terminal pro–B-type natriuretic peptide, ETA: endothelin type A, ETB: endothelin type B, CKD: chronic kidney disease, SERAPHIN: study with the endothelin receptor antagonist in pulmonary arterial hypertension to improve clinical outcome, AMBITION: Ambrisentan and Tadalafil in patients with pulmonary arterial hypertension, CTEPH: chronic thromboembolic pulmonary hypertension, LFT: lever function test, PDE5I: phosphodiesterase 5 inhibitors.

Name of the article	Name of the first author	Year of publication	Journal Name	Summary
Respirable controlled release polymeric colloid (RCRPC) of Bosentan for the management of pulmonary hypertension: in vitro aerosolization, histological examination, and in vivo pulmonary absorption [1]	Lydia A. Hanna	2017	Drug Delivery	Bosentan-loaded RCRPC showed improved bioavailability and reduced adverse effects compared to oral dosages. So, bosentan-loaded RCRPC delivered via the pulmonary route can advance PAH treatment [1].
Comparative efficacy and acceptability of endothelin receptor antagonists for pulmonary arterial hypertension: a network meta-analysis [2]	Mi-Ma Duo-Ji	2017	International Journal of Cardiology	It recommended that Ambrisentan is an influential ERA based on improving 6MWD, safety, efficacy, and clinical status deterioration [2].
Pulmonary arterial hypertension epidemiology and registries [3]	Michael D. McGoon	2013	Journal of the American College of Cardiology	Registries are critical in refurbishing knowledge of PAH, and since the pioneer US National Institutes of Health (NIH) Patient Registry for Primary Pulmonary Hypertension (PPH Registry), the national and international registries noted changes in PAH phenotypes and outcomes and helped in the management protocols [3]
Pulmonary arterial hypertension: Diagnosis, treatment, and novel advances [4]	Bradley A. Maron	2021	American journal of respiratory and critical care medicine	The pivotal goal of PAH ranged from delaying mortality to the restoration of normal right ventricular function and structure, and recent data provided an evidence-based framework for an individualized- patient-centered approach of both pharmacological and non-pharmacological approaches for PAH management [4]
Diagnosis and treatment of pulmonary arterial hypertension a review [5]	Nicole F. Ruopp	2022	Jama	If untreated, PAH patients typically progress to heart failure and death, while first-line targeted combination drug therapies provide improved survival outcomes [5].
Treatment of pulmonary arterial hypertension with the dual endothelin receptor antagonist macitentan: clinical evidence and experience [6]	Catharina Belge	2019	Therapeutic Advances in Respiratory Disease	Macitentan is the first drug with long-term outcome efficacy in PAH treatment with improved WHO FC and less hepatotoxicity than Bosentan and less edema than Bosentan and Ambrisentan, with some uncertainty in pediatric effectiveness [6].
Pulmonary arterial hypertension [7]	David Montani	2013	Orphanet journal of rare diseases	Although massive advancements in PAH treatment took place still, no curative treatment is available, and the prognosis is still poor [7]
The molecular targets of approved treatments for pulmonary arterial hypertension [8]	Marc Humbert	2016	Thorax	The usefulness of combination therapy is still inconclusive, and PAH is still not curable despite the recent medical advancements that need new treatments [8].
Ambrisentan for the treatment of pulmonary arterial hypertension: improving outcomes [9]	Soha M Elshaboury	2013	Patient Preference and adherence	Ambrisentan has been proven beneficial in attenuating the clinical worsening rate and increasing exercise capacity with some SEs of peripheral edema, headache, sinusitis, and potential risk of teratogenicity [9].
Therapies for pulmonary arterial hypertension: where are we today, where do we go tomorrow? [10]	Andrei Seferian	2013	European Respiratory Review	Though PAH is still an incurable disease with a worse prognosis, the last 20 years have tremendously advanced new therapies such as selexipag, Macitentan, and riociguat with a safer profile. Still, it will be challenging to supplant the older drugs with new ones in terms of efficacy [10].
Management of pulmonary arterial hypertension [11]	Vallerie V. McLaughlin	2015	Journal of the American College of Cardiology	The timely diagnosis and differentiation of PAH from pulmonary vascular hypertension (PVH) are crucial. Right ventricular health is the key prognostic factor that may pave the way for active research and translational discoveries [11].
Pharmacologic treatments for pulmonary hypertension: exploring pharmacogenomics [12]	Julio D Duarte	2013	Future Cardiology	WHO group 1 PAH is the least common, but surprisingly, most research is on this group. In contrast, more research is necessary on groups like WHO groups 2, 3, 4, or 5. It yielded three drug classes such as PDE5 Is, prostacyclin analogs, and ERAs, and no treatment proved to be ideal except prostacyclin analog with mortality benefit [12].
The transition From Ambrisentan to Macitentan in patients with pulmonary arterial hypertension: a real-world prospective study [13]	Yusi Chen	2022	Frontiers in Pharmacology	The transition from Ambrisentan to Macitentan to a selected cohort showed that the change is safe and improved exercise capacity, NT-pro BNP, and proper ventricular function with few SE like menstrual disorder and anemia which may necessitate monitoring and interventions in younger patients [13]
Systematic review of randomized controlled trials of endothelin receptor antagonists for pulmonary arterial hypertension [14]	Michael Kuntz	2016	Lung	ERA reduces hospitalization rate and disease progression and improves long-term morbidity and mortality; newer agents showed favorable toxicity profiles with better hemodynamics [14].
The current state of endothelin receptor antagonism in hypertension and pulmonary hypertension [15]	Kazuya Miyagawa	2014	Therapeutic advances in cardiovascular disease	The ETA receptor antagonism is pivotal in hypertension treatment. Due to its pathophysiology, both ETA and ETB receptor blocking is necessary for PAH treatment, so dual ERA, like Macitentan, might be the appropriate drug for PAH treatment [15].
Pathways and drugs in pulmonary arterial hypertension – focus on the role of endothelin receptor antagonists [16]	Rosalinda Madonna	2015	Cardiovascular drugs and therapy	As per the available randomized clinical trials, Macitentan improved clinical outcomes compared to Bosentan and Ambrisentan, but long-term prognosis data are still not acceptably available [16]. Lung transplantation planning and combination therapeutic approaches should be the treatment plan in patients with progressive symptoms despite medical therapies [16].
Clinical Pharmacology of endothelin receptor antagonists used in the treatment of pulmonary arterial hypertension [17]	Marie-Camille Chaumais	2015	American Journal of Cardiovascular Drugs	Available data evidenced that dual and specific ERAs effectively improve hemodynamics, functional status, and exercise capacity; in several randomized placebo-controlled trials [17].
Endothelin-receptor antagonists beyond pulmonary arterial hypertension: cancer and fibrosis [18]	John-David Aubert	2016	Journal of medicinal chemistry	Though there have always been debates about the drug to choose from ETA and ETB receptor selectivity for PAH treatment, presently ETA selective and dual ETA and ETB selective antagonists are in use, and ETA particular drugs showed more fluid retention compared to dual ETA and ETB receptors even in fibrosis-related disorders [18]. In conclusion, combination therapy is beneficial in achieving maximum efficacy, and although not curative, these drugs are helpful with tolerable SEs [18].
Endothelin receptor antagonists: Status quo and future perspectives for targeted therapy [19]	Frederik C. Enevoldsen	2020	Journal of Clinical Medicine	The ERAs are beneficial for PAH treatment, evidenced by improved clinical outcomes, and they could be a promising treatment for chronic kidney disease (CKD), cancer, and pain management [19].
Endothelin-receptor antagonists in the management of pulmonary arterial hypertension: where do we stand? [20]	Michele Correale	2018	Vascular health and risk management	Promising results came from SERAPHIN (Study with the Endothelin Receptor Antagonist in Pulmonary Arterial Hypertension to Improve clinical outcome) and AMBITION (Ambrisentan and Tadalafil in Patients with Pulmonary Arterial Hypertension) studies, and less favorable results came from the COMPASS-2 survey, which needs clarification. Still, it may not be associated with class effects but may signify the impact of specific drugs [20].
Clinical pharmacokinetics and pharmacodynamics of the endothelin receptor antagonist macitentan [21]	P. N. Sidharta	2015	Clinical Pharmacokinetics	This article discussed some studies which reinforce the use of Macitentan concerning tolerability and efficacy compared to other approved ERAs [21].
Long-term results from the EARLY study of Bosentan in WHO functional class II pulmonary arterial hypertension patients [22]	Gérald Simonneau	2014	International journal of cardiology	The four-year survival rate for WHO FC II was about 85%, and there was increased mortality risk in patients with a low mixed venous oxygen saturation, 6MWD, and a high NT-proBNP level [22].
Bosentan therapy for pulmonary arterial hypertension and chronic thromboembolic pulmonary hypertension: a systemic review and meta-analysis [23]	Xinwang Chen	2018	The clinical respiratory journal	Bosentan is efficacious in preventing exacerbations and improving hemodynamics and exercise capacity in PAH patients [23]. CTEPH patients showed no significant changes, and oral Bosentan is safe, with a possible risk of abnormal LFT [23].
Evaluation of efficacy, safety, and tolerability of Ambrisentan in Chinese adults with pulmonary arterial hypertension: a prospective open-label cohort study [24]	Y. Huo	2016	BMC Cardiovasc Disord	Ambrisentan showed similar efficacy and safety in the Chinese population regarding improvement of exercise capacity and prevention of clinical worsening in 5 mg and 10 mg doses, and it could be a valuable approach for Chinese people with PAH [24].
Efficacy, safety, and clinical pharmacology of Macitentan in comparison to other endothelin receptor antagonists in the treatment of pulmonary arterial hypertension [25]	Jasper Dingemanse	2014	Expert opinion on drug safety	Macitentan showed better efficacy in both treatment-naïve patients and patients with other PAH therapy in terms of safety, tolerability with low drug interaction rates, and no need for dose adjustment in chronic liver or kidney disease patients compared to Bosentan and Ambrisentan [25].
SERAPHIN hemodynamic substudy: the effect of the dual endothelin receptor antagonist macitentan on hemodynamic parameters and NT-proBNP levels and their association with disease progression in patients with pulmonary arterial hypertension [26]	Nazzareno Galie	2017	European heart journal	By improving hemodynamics and reducing NT-proBNP level, Macitentan improved the chance of threshold value reaching and reducing morbidity and mortality irrespective of WHO FC and background PAH-specific therapy [26].
Macitentan and morbidity and mortality in pulmonary arterial hypertension [27]	Tomás Pulido	2013	N Engl J Med	Mcicentan notably decreased morbidity and mortality in previously and never-treated [27].
Updated treatment algorithm of pulmonary arterial hypertension [28]	Nazzareno Galie	2013	Journal of the American College of Cardiology	This article demonstrated the stepwise treatment algorithm of PAH based on pieces of evidence from the last five years and European Society of Cardiology grades of recommendation [28].
Upfront triple combination therapy in pulmonary arterial hypertension: a pilot study [29]	Olivier Sitbon	2014	European Respiratory Journal	This small population pilot study was not a randomized controlled trial, so results cannot be directly compared to monotherapy or combination therapeutic approaches for PAH patients. Still, it showed some preliminary evidence to support the long-term efficacy of upfront triple combination therapy [29].
The role of combination therapy in managing pulmonary arterial hypertension [30]	Hossein-Ardeschir Ghofrani	2014	European Respiratory Review	The long-duration randomized controlled trials provided evidence in favor of combination therapies for PAH, which may influence physician's choice of first-line treatments in the future [30].
Pulmonary arterial hypertension: Pathophysiology and treatment [31]	Norris S. H. Lan	2018	Diseases	The currently available five drug classes, namely PDE5Is, soluble guanylate cyclase inhibitors, prostacyclin analogs, prostacyclin receptor agonists, and ERAs, have been proven as beneficial as initial therapy either as monotherapy or in combination in recent long-term trials evidenced by progression-free survival of PAH patients [31].

Discussion

We will discuss the endothelin receptor's (ER) effects from different aspects like the endothelin pathway and its role in developing pulmonary hypertension, the pharmacology of ERAs, the efficacy, and safety of ERAs, combined with some study result findings. We will further connect the studies' crucial conclusions and endpoints, along with our suggestions for the scope of future studies.

Endothelin and Its Role in PAH

According to Kuntz et al., endothelins are composed of 21 amino acid peptides, which have a vast range of physiological actions such as vasomotor tone modulation, the proliferation of cells, and the production of hormones [14]. There are different sources of endothelins, such as vascular endothelial cells, which are also considered the primary source and produce all types of endothelins (endothelin types 1, 2, and 3). Besides these cells, endothelial cells produced by vascular smooth muscle cells and other cells, including macrophages, cardiac muscle cells, glial cells, fibroblasts, and glomerular mesangial cells, have endothelin type 1 [14]. Endothelin production starts with producing an inactive precursor named pre-pro-endothelin-1 (ET-1), which later divides into larger ET-1; finally, endothelin-converting enzymes (ECEs) cleave the more significant endothelin into its biologically active form. It is secreted as a paracrine hormone and regulates vascular tone as the most critical regulator [14]. The authors described it as a potent smooth muscle mitogen overexpressed in idiopathic PAH, which is under group 1 of the PAH classification [14]. As described by Miyagawa and Emoto, the endothelin receptors act at autocrine/paracrine levels, as evidenced by the fact that ET-1 is secreted as a preliminary luminary in the vessel wall. Also, the ET-1 plasma concentration is lower than the threshold required for activating the ET-1 receptors [15]. As per Madonna et al., almost 80% of ET-1 is secreted basolaterally into the pulmonary interstitial tissue instead of being apically secreted into the bloodstream [16]. Table 2 shows the PAH classification as described in [6,10].

**Table 2:** Pulmonary arterial hypertension clinical classification As described in [6,10]. BMPR2: bone morphogenetic protein receptor type-2, HIV: human immunodeficiency virus, PAH: pulmonary arterial hypertension.

Group 1. Pulmonary arterial hypertension	
1.1 Idiopathic PAH	
1.2 Heritable PAH	1.2.1 BMPR2 mutation
	1.2.2 Other mutations
1.3 Drug and toxin-induced	
1.4 Associated with	1.4.1 Connective tissue disease
	1.4.2 HIV infection
	1.4.3 Portal hypertension
	1.4.4 Congenital heart diseases
	1.4.5 Schistosomiasis
Group 1'. Pulmonary venous-occlusive disease or pulmonary capillary hemangiomatosis	
Group 1". Persistent pulmonary hypertension of the newborn	
Group 2. Pulmonary hypertension due to left heart disease	2.1 Left ventricular systolic dysfunction
	2.2 Left ventricular diastolic dysfunction
	2.3 Valvular disease
	2.4 Congenital/acquired left heart inflow/outflow tract obstructions and congenital cardiomyopathies
	2.5 Congenital/acquired pulmonary veins stenosis
Group 3. Pulmonary hypertension due to lung diseases or hypoxia	3.1 Chronic obstructive pulmonary disease
	3.2 Interstitial lung disease
	3.3 Other pulmonary diseases with a mixed restrictive and obstructive pattern
	3.4 Sleep-disordered breathing
	3.5 Alveolar hypoventilation disorders
	3.6 Chronic exposure to high altitude
	3.7 Developmental lung diseases
Group 4. Chronic thromboembolic pulmonary arterial hypertension and other pulmonary artery obstruction	4.1 Chronic thromboembolic pulmonary hypertension
	4.2 Other pulmonary artery obstructions: 4.2.1 angiosarcoma; 4.2.2 other intravascular tumors; 4.2.3 arteritis; 4.2.4 congenital pulmonary arteries stenoses; 4.2.5 parasites (hydatidosis)
Group 5. Pulmonary hypertension with unclear or multifactorial mechanisms	

Endothelin-1 works through two receptors that are G protein-coupled and named endothelin A (ETA) and endothelin B (ETB), which are found on vascular smooth muscle cells and endothelial cells, respectively [14]. While ETA mediates vasoconstriction and proliferation of cells, ETB is found mainly on endothelial cells and mediates the release of nitric oxide (NO) and prostaglandin (PG). Despite being present in vascular smooth muscle cells, ETB does not play a significant role in vasoconstriction [14]. As described by Miyagawa and Emoto, all endothelin receptors activate the intracellular pathways, consisting of the increasing influx of calcium, phospholipase Cβ activation, activation of protein kinase C, phospholipase D, mitogen-activated protein kinases, and the Rho/Rho kinase pathway [15]. ET-1 has a ten times higher affinity than endothelin-2 (ET-2), while endothelin-3 (ET-3) has the lowest relationship [15].

Chaumais et al. described ET-1 as one of the potent constrictors with more prolonged pharmacological effects that, along with vasoconstriction that promotes collagen deposition, fosters the proliferation of cells and inflammation [17]. According to Aubert et al., Furins and ECE activate [18]. Furins are a group of intracellular serine proteases belonging to the subtilisin-like proprotein convertase family that process latent precursor proteins on amino acid paired sequences (Arg/Lys-Arg) to biologically active forms [18]. These endogenous proteases can cleave other precursor proteins, such as precursor polypeptides of 212 aa (ppETs), and are associated with several human disorders [18]. The primary location of Furins is in the Golgi/trans-Golgi secretory pathway, and the catalytic mechanism of this serine protease family is a triad of essential amino acids, including one histidine, one aspartic acid, and one serine [18]. As per the authors, two metalloprotease ECEs are in existence, such as 1,9−12 ECE-1 (EC 3.4.24.71), which hydrolyzes the Trp11-Val12 bond of human big-ET-1 when it is at a neutral pH state and has four isoforms [18]. There is another one named ECE-1 (EC 3.4.24.71) that hydrolyzes the Trp11-Val12 bond of human big-ET-1 again at a neutral pH state, and it has four isoforms, such as ECE-1a−d, which acts from one gene by the use of different protomers [18]. ECE-2 is another one that works at an acidic pH, but its information about human cells' physiological processes is insufficient [18]. According to the authors, ECE-1 can be secreted or expressed at the surface of the cells (ECE-1a and ECE-1c), so they are accessible to inhibitors. At the same time, ECE-1 can remain intracellular (ECE-1b and ECE-1d) and transverse the cell membrane [18]. Neutral+endopeptidase 24.11 (NEP24.11)/neprilysin can also activate and break big ET-1 but with lower affinity than ECE-1.15, while mast-cell-derived chymase can degrade the Tyr31-Gly32 bond of bigger ET-1 to yield ET-1 (1-31) [18]. The peptide is processed further into active ET-(1-21) by ECE-1, the principal activator of big ET-1, or NEP24,11 [18].

According to Miyagawa and Emoto, ETB mediates the clearance of endothelin, which undergoes intracellular internalization and degradation [15]. The half-life of ET-1 is rapid (less than 60 seconds), and it clears through pulmonary circulation [15]. The authors also described that a crosslink might be there between ETA and ETB, and these may form functionally active heterodimers, which quality of ERs can create reasoning on how we can block the endothelin (ET) system with ERAs most effectively for the treatment purpose [15]. 

Architectural Structures of Endothelin Receptor Antagonists

According to Chaumais et al., after the invention of ET-1 in 1988, the ERAS was discovered as a dual endothelin receptor antagonist that acts on ETA and ETB receptors based on the pyramidic sulfamide class of the Ro 46-2005 molecule [17]. In 1991, the scientists invented Ro 47-0203 (Bosentan) through structural optimization for improved pharmacokinetic/pharmacodynamic properties [17]. Bosentan is the first approved ERA for PAH, a non-peptide pyrimidine derivative that competitively and specifically inhibits ET-1 receptor binding to both ETA and ETB subtypes of receptors, resulting in irreversible blockage of their activities [17].

A highly selective ETA receptor antagonist Ambrisentan was developed depending on the theoretical fact that selective ETA receptor blocking has benefited by selectively preventing ETA-induced vasoconstriction and cellular proliferation while conserving vasodilation and clearance functions of ETB [17].

**Figure 1:**
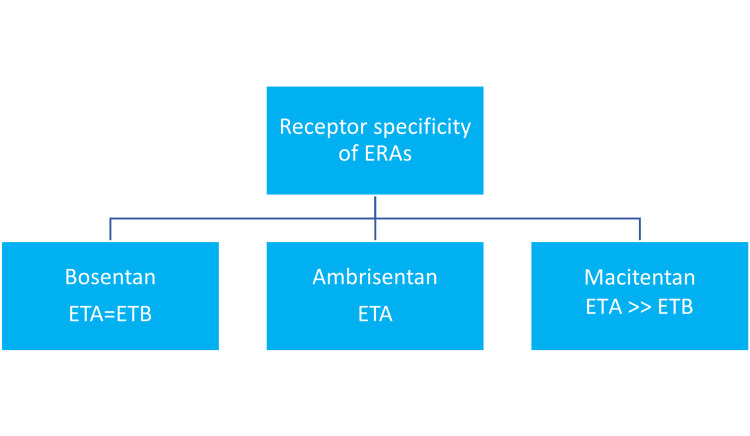
Receptor affinity of endothelin receptor antagonists ERA: endothelin receptor antagonist, ETA: endothelin receptor type A, ETB: endothelin receptor type B

The newest non-peptide drug, Macitentan, was approved by European Medicines Agency (EMA) and the FDA for long-term management of PAH, which has high tissue targeting with sustained receptor binding and 50-fold higher affinity for ETA than ETB receptors [17]. Figure 1 displays the receptor selectivity of the ERAs.

Pharmacology of Endothelin Receptor Antagonists

According to Aubert et al., as ERAs are therapeutic options for chronic diseases of fibrogenic and hypertensive disorders, they necessitate drug intake once or twice a day with high oral bioavailability for the patient to comply with the treatment [18]. As these drugs have nanomolar/picomolar half-maximal inhibitory concentration (IC50) values for ET-1 binding to its corresponding receptors, the receptor antagonists also have to be of a similar level of nanomolar or picomolar affinity [18]. As a result, the products developed as therapeutic ERAs should have high oral bioavailability and receptor affinity [18]. Researchers started working on developing ERAs, and many synthetic molecules already have beyond design, preparation, and evaluation in both in vitro cellular models and in vivo experimental lab animal models of human diseases and eventually in human clinical trial stages [18]. The authors also mentioned that some compounds are already on the market for PAH treatment, and late clinical trials have evaluated some other blends for different diseases. At the same time, the molecules were orally bioavailable and were active at even nanomolar blood concentrations [18]. The design of these molecules was in such a way that they had to be either ETA-selective antagonists with a selectivity ETA/ETB of more than 100 or dual ETA/ETB antagonists with selectivity almost similar for ETA and ETB, as well as some rarely produced molecules with ETB selective antagonists with a selectivity ETA/ETB less than 100. After clinical evaluation of all these molecules, only two types of ERAs reached the level of clinical use: ETA-selective antagonists and dual antagonists [18,19]. According to Correale et al., the ETA receptor selectivity is 20, 50:1, and 200:1 for Bosentan, Macitentan, and Ambrisentan, respectively [20]. However, the selective ETA receptor antagonists work as dual ETA/ETB antagonists at high concentrations [18].

Pharmacokinetics and Pharmacodynamics of Endothelin Receptor Antagonist

Endothelin receptor antagonists have different pharmacokinetic and pharmacodynamic properties and receptor affinity, which will be discussed separately for the approved ERAs in this review section. According to Chaumais et al., several animal model studies showed the effectiveness of both selective and non-selective ERAs in PAH, which is done by prompting pulmonary vasodilation and decreasing pulmonary vascular remodeling and right ventricular hypertrophy [17].

Pharmacokinetics and Pharmacodynamics

Bosentan: According to Enevoldsen et al., Bosentan is the first approved drug for PAH treatment for WHO functional classes III-IV, a non-peptide pyrimidine derivative [19]. As described by Chaumais et al., Bosentan for adults and pediatric populations has similar pharmacokinetic properties. However, the pharmacokinetics of Bosentan was only studied in healthy people as an oral drug with a 125 mg twice-daily adult dose followed by four weeks of titration to 62.5 mg twice-daily [17]. Bosentan tends to have a dose-dependent proportional pharmacokinetic increase of up to 500 mg daily. Still, higher doses have less proportional pharmacokinetics for both maximum concentration (C_max_) and area under the curve (AUC) [17]. The absolute bioavailability of Bosentan is 50%, which reaches peak plasma concentration after three hours and steady-state concentration after three to five days when multiple doses are administered [17,19]. The volume of distribution is approximately 30 L, with a clearance of 17 L/h. Any dose adjustment is unnecessary for adults based on age, sex, race, or even body weight for this highly albumin-bound drug [17]. Bosentan is mainly metabolized by hepatic cytochrome P450 (CYP) 2C9 and 3A4; less than 3% of an oral dose is excreted in urine [17]. Although severe renal impairment does not interfere with Bosentan's pharmacokinetics, severe liver disease is a contraindication [17].

As described by Chaumais et al., Bosentan decreases the pulmonary arterial pressure rise by preventing pulmonary vascular remodeling and reducing right ventricular hypertrophy in chronic hypoxia or monocrotaline (MCT)-induced pulmonary hypertension models. Bosentan inhibits collagen deposition in the RV in chronic hypoxia-exposed rats. It also attenuates the inflammatory response and arterial smooth muscle cell proliferation in guinea pigs and mouse lungs [17]. Additionally, Bosentan reduces intracellular adhesion molecule (ICAM-1) and plasmatic IL-6 levels in humans, which correlates to hemodynamic improvement [17].

Ambrisentan: As described by Enevoldsen et al., Ambrisentan is a structurally propionic acid derivative [19]. According to Elshaboury and Anderson [9] and Enevoldsen et al. [19], Ambrisentan is a drug with unknown bioavailability that is rapidly absorbed without food interference and reaches its peak two hours after administration. This high protein-bound [99%] drug undergoes intestinal extrusion by the P-glycoprotein system, and less than 5% is renally excreted [9]. There is no need for dose adjustment for Ambrisentan in mild to moderate renal impairment patients, though no current data are available on using Ambrisentan in severe renal disease or hemodialysis patients [9]. Ambrisentan uses cytochrome P450 [CYP]3A4 and CYP2C1, as well as the uridine 5′-diphosphate glucuronosyltransferases 1A9S, 2B7S, and 1A3S for metabolism, according to the authors [9].

Ambrisentan reduces pro-inflammatory gene expression in ischemia/reperfusion models and thus plays a cytoprotective role in vascular microcirculation. Contrary to other ERAs, it has no experimental data regarding its use in pulmonary hypertension, as described by Chaumais et al. [17].

Macitentan: Enevoldsen et al. described Macitentan as a sulfonamide drug class [19]. According to Chaumais et al. and Enevoldsen et al., the bioavailability of Macitentan is unknown, and it has inhibitory potency on both ER and its high lipophilicity leads to its dose-proportional slow absorption in an aqueous environment [17,19]. Macitentan has a median time to reach the maximum (t_max_) of approximately eight hours and a half-life of 17.5 hours at a one-day 300 mg dosage [17]. In contrast to Bosentan and Ambrisentan, this drug excretes through the kidneys. According to the authors, two prospective, single-center, open-label studies compared Macitentan's pharmacokinetics in healthy and severely hepatic or renally impaired patients and eventually reported no clinical relevance or dose adjustment requirement [17].

As Macitentan is a competitive ERA with slower receptor dissociation kinetics than Ambrisentan and Bosentan, it has enhanced pharmacological activity in PAH, as Chaumais et al. [17]. In vivo, Macitentan has the highest receptor binding among all 3 ERAs, and in PH rats, Macitentan increases survival by attenuating pulmonary arterial pressure and subsequent right ventricular hypertrophy while escaping any effect on systemic blood pressure [17].

Efficacy and Safety of Endothelin Receptor Antagonists

**Figure 2:**
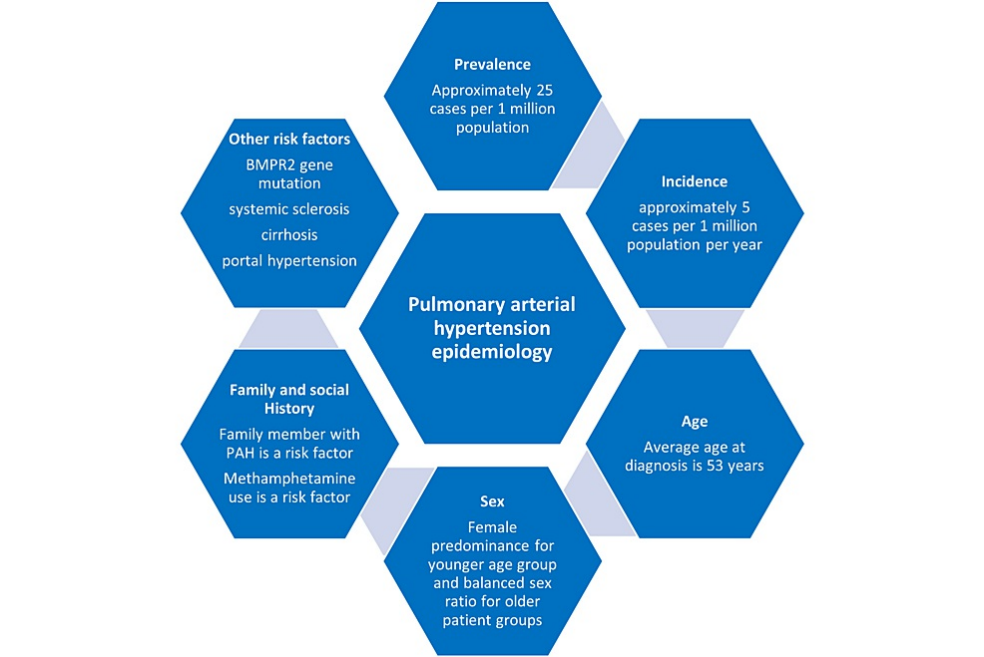
Epidemiology of pulmonary arterial hypertension BMPR2: bone morphogenetic protein receptor type 2, PAH: pulmonary arterial hypertension Reference: [3]

**Figure 3:**
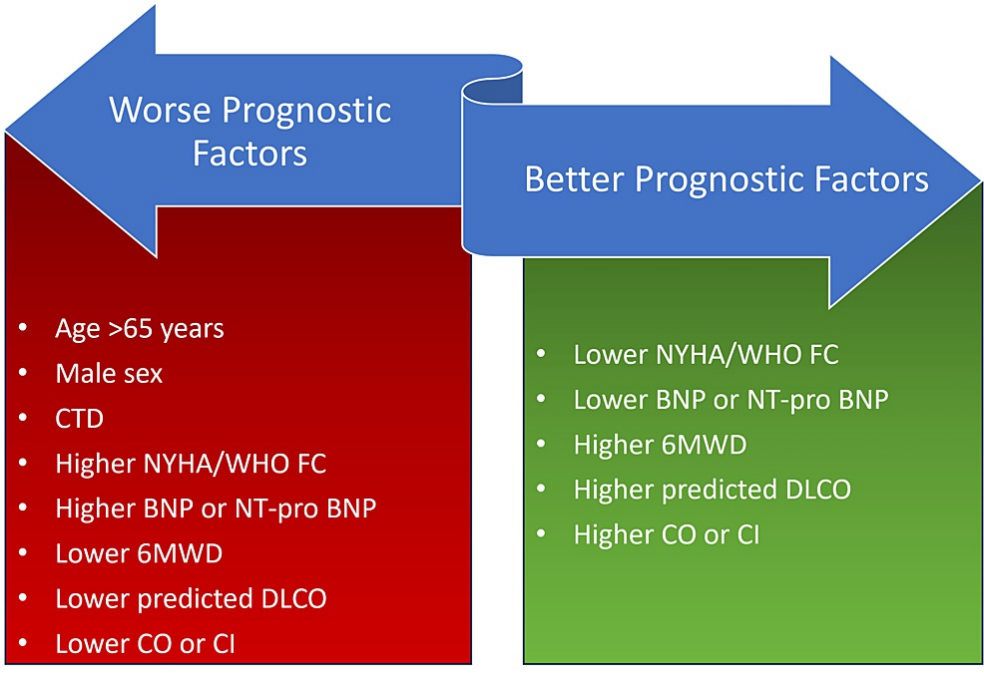
Prognostic factors of PAH CTD: connective tissue disease, NYHA: New York Heart Association, WHO FC: world health organization functional class, BNP: B-type natriuretic peptide, NT-pro BNP: N-terminal pro-B-type natriuretic peptide, 6MWD: six minutes walking distance, DLCO: diffusing capacity of lung for carbon monoxide, CO: cardiac output, CI, cardiac index. Reference: [3]

This section will discuss the efficacy and safety profiles of endothelin receptor antagonists. According to Sidharta et al., the ERAs were approved based on 12-16 weeks of short-term studies with a smaller group of participants and only based on improved exercise capacity. However, recent guidelines recommend long-term outcome studies to evaluate ERA's efficacy and safety [21]. This is due to the fact that the prognosis of PAH depends on several factors, including its epidemiology. We will discuss each endothelin receptor's efficacy and safety profile separately while pooling information from the latest studies on these drugs as monotherapy and combination therapy. We have shown the epidemiology and prognostic factors in Figures 2-3 [3], respectively.

Bosentan: According to Simonneau et al., the only randomized control trial on Bosentan to date is the double-anonymized phase of the early study of Bosentan on WHO FC II patients [22]. The WHO FC comprises four classes based on severity, from least to most severe: classes I-IV [22]. This study is exclusively focused on the WHO functional class II pulmonary hypertension population.

According to Simonneau et al., the study was a double-blinded, randomized, multicenter study of 185 patients from 21 countries who received either Bosentan or placebo [22]. At the end of 51 months of median exposure to Bosentan, 77.8% of patients were in WHO FC I/II, while 20.2% discontinued the drug over this period due to adverse effects. The principal negative impact was aminotransferase elevation to more than three times the upper limit of normal in 16.8% of the study population [22]. The factors associated with a higher mortality risk were low 6MWD and mixed venous oxygen saturation, high N-terminal prohormone of brain natriuretic peptide levels, and connective tissue disease-associated PAH patients [22]. The survival rate was 84.8 (95% CI 79.4, 90.2), and the PAH event-free survival rate was 79.5% (95% confidence intervals [95% CI] 73.4, 85.6) [22]. As described by the authors, the main limitations of this study were the lack of a comparator, the not using a long-term placebo arm due to ethical reasons, and some patients were already taking other drugs, such as sildenafil, at baseline [22]. In a nutshell, the majority of the patients included in this study had improved or maintained their functional class with long-time Bosentan exposure, and about 20% of the patients discontinued using Bosentan due to some SEs such as elevated hepatic aminotransferases or worsening of PAH [22]. According to Correale et al., because of Bosentan's hepatotoxicity risk, the US Food and Drug Administration recommended monthly liver function tests and every three-monthly blood test for anemia [20].

According to a systematic review and meta-analysis performed by Chen et al., 10 RCTs comprised of 1185 patients with both PAH and chronic thromboembolic pulmonary hypertension (CTEPH) showed reduced mean pulmonary arterial pressure, prolonging 6MWD, increased cardiac index, reduced pulmonary vascular resistance, and prevented functional class deterioration of PAH, increasing the risk of abnormal LFT in both PAH and CTEPH patients [23]. There need to be more studies to evaluate the efficacy and safety of Bosentan in our opinion.

Ambrisentan: As described by Elshaboury and Anderson, Ambrisentan is an approved drug for WHO class 1 PAH patients and the ARIES series (Ambrisentan for the treatment of pulmonary arterial hypertension, randomized, double-blind, placebo-controlled, multicenter efficacy studies) [9].

The ARIES series consists of ARIES-1 and ARIES-2 trials, ARIES E, and ARIES-3 studies. The phase III ARIES-1 and ARIES-2 trials were conducted simultaneously between December 2003 and February 2006 and enrolled 202 and 192 class 1 PAH patients, respectively [9]. This study included patients with class 1 PAH and excluded patients taking other PAH medications [9]. After the ARIES-1 and ARIES-2 studies, an extension study was conducted, which included patients who completed previous ARIES studies but dropped out due to initial exclusion criteria but were eventually able to continue ambrisentan monotherapy for two consecutive years, consisting of a total of 383 patients [9]. The final study of the ARIES series was an open-label, long-term, single-arm, multicenter ARIES-3 study, which included a total of 224 patients with WHO groups 1 to 5, including patients who were taking other PAH medications and had failed therapy with Bosentan or sitaxsentan due to raised aminotransferase elevation more than three times the upper limit of the standard (the ARIES-3 study included patients with aminotransferase levels less than three times the upper limit of the normal during screening) [9].

The combined outcome of the ARIES series showed that Ambrisentan is effective both as monotherapy and as a combination therapy with other PAH medications. It increases exercise capacity, reduces FC deterioration risk, and stabilizes the disease [9]. The authors also described that Ambrisentan has a mortality benefit with an 88% survival rate after two years and tolerability in most patient groups with few SEs, including headache, sinusitis, nasal congestion, peripheral edema, and flushing [9]. Ambrisentan is preferable to Bosentan due to additional benefits such as once-daily dosing, minor adverse effects, fewer drug interactions, a low risk of aminotransferase elevation, and so on. However, as per the authors, further clinical trials are required to determine the absolute use of Ambrisentan for PAH treatment [9].

As described by Huo et al., an open-label, single-arm, phase IIIb study was conducted at 12 Chinese centers from December 2012 to August 2014 on 133 patients, where Ambrisentan significantly improved exercise capacity, decreased NT-pro-BNP plasma level, and decreased the Borg Dyspnoea Index (BDI) from baseline [24]. Approximately 34.3% of the study population showed adverse drug effects, most commonly peripheral edema and flushing in 11.2% and 8.2% of patients, respectively [24].

Macitentan: As described by Dingemanse et al., the double-blinded, placebo-controlled, randomized, parallel-group, event-driven phase III study named SERAPHIN was conducted to evaluate the long-term effects of Macitentan on the morbidity and mortality risks of PAH patients [25]. This study comprised 742 patients of different types who were between 12 and 84 years of age and must have a 6MWD of more than or equal to 50 minutes and be in WHO functional class II to IV to get into this study [25, 26]. The investigation started by screening and randomizing with 6MWD and WHO FC at the start and then at months 3, 6, and every six months till the end of the treatment period [25]. As described by Pulido et al., the intention-to-treat method analyzed the study endpoints, which comprised all the randomized patients, and the Kaplan-Meier method and log-rank test were used to estimate all time-to-event endpoints [27].

As described by Galie et al., Macitentan showed improved outcomes compared to placebo in both 3 mg and 10 mg doses regarding mean pulmonary arterial pressure (mPAP), pulmonary vascular resistance (PVR), and NT-proBNP levels regardless of WHO FC and background PAH-specific therapy [26]. Macitentan proved superior in hepatic and edema safety profiles compared to Bosentan and Ambrisentan and similar in the safety profile of hemoglobin level reduction [20,25]. As described by Sidharta et al., Macitentan is not an active drug transporter substrate, so it is possibly a safer drug for the liver [21]. This drug has fewer drug interactions and does not necessitate dose adjustment in patients with hepatic or renal impairment [25].

Other Treatment Options for Pulmonary Arterial Hypertension

Apart from ERAs, some other treatment options are available for PAH treatment. As described by McLaughlin et al. and Galiè et al., the PAH treatment starts with general health measures starting with moderate exercise, psychological support, vaccination, a salt-restricted diet, and maintaining oxygen saturation >90% both at exertion and at rest [11,28]. Apart from targeted therapies, some classes of drugs work as background drugs, such as diuretics, anticoagulants, calcium channel blockers, digitalis, and beta blockers for treatment [7,11]. Other available treatment options can be surgical, such as lung transplantation with extracorporeal life support and balloon atrial septostomy, though treatment options should be highly individualized for every patient [7,11]. As described by Farber and Loscalzo, despite these advancements in treatment options, there is still no curative disease with a good PAH prognosis [7].

Combination Therapy for Pulmonary Arterial Hypertension

As described by Sitbon et al. and Ghofrani et al., pulmonary hypertension is a progressively devastating disease with a complex pathogenesis involving multiple pathways, including endothelin, prostacyclin, and the nitric oxide pathway as the principal ones, so there is scope for combination therapy for PAH treatment [29,30]. The combination treatment, named intensification from monotherapy, can be done from the start while using two or three drugs in a treatment-naïve patient and is called upfront therapy [30]. Additionally, combining two or three pills, either as first-line therapy or sequential therapy, also helps patients with other cardiovascular or hypertensive complaints, as per Humbert et al. [8]. The authors mentioned a pilot study with a retrospective data collection strategy from a prospective registry of newly diagnosed NYHA FC III/IV patients (n=19) who were started upfront on combination therapy of intravenous (IV) epoprostenol, Bosentan, and sildenafil [29]. Among the 19 patients, the 6MWD and hemodynamics improved in 18 patients, and NYHA FC to I or II improved in 17 patients at month 4 [29]. One patient was excluded at month 4 from the study due to an emergency lung transplantation at month 3, and finally, all 18 patients showed clinical and hemodynamic improvement [29]. Though the French equation calculated expected survival at 75% (95% CI 68-82%), 60% (95% CI 50-70%), and 49% (95% CI 38-60%) at one, two, and three years, respectively, it was finally 100% for the consecutive three years [29]. So, this pilot study provides the initial proof of the long-term efficacy of upfront triple-drug therapy in severe PAH patients [29].

Lan et al. [31] described a few studies regarding PAH combination therapies, such as a meta-analysis by Galie et al. [28] that revealed clinical improvement, a reduction in mortality, and a reduction in time to clinical worsening (TTCW) with sequential combination therapy. Still, this study lacked significant statistical power, although a meta-analysis later proved combination therapy superior to monotherapy.

Ghofrani and Humbert [30] and Lan et al. [31] also described three more studies named the Bosentan randomized trial of endothelin antagonist therapy (BREATHE-2) for PAH study, which was an extension of the BREATHE-1 study and was the first to evaluate combination therapy. Still, it had a small sample size of 33 and failed to illustrate significant differences in clinical outcomes [31]. A further essential study was AMBITION [31]. This study investigated the role of dual therapy in treatment-naive patients for 73 weeks, randomized to either combination therapy or monotherapy with tadalafil or Ambrisentan, where both groups showed a targeted clinical endpoint, which was time to a clinical failure event, and dual treatment provided a further 50% relative reduction [8,31].

## Conclusions

In this review article, we discussed the role of endothelin receptor antagonists in treating pulmonary arterial hypertension. Three main ERAs are currently in use, and all ERAs oppose the endothelin-induced pathogenesis of PAH. Some studies evaluated all these drugs as both monotherapy and combination therapies. They all proved clinically effective in improving disease outcomes in PAH patients with some SEs, most commonly hepatotoxicity and worsening of PAH status. In our article, we combined the pharmacological properties and results from the available studies, which may pave the way to focusing more on the scope of PAH treatments. We included reports from the last ten years in the English language and free full texts only, which may be a limitation of this study. We recommend further research on these drugs as monotherapy and combination therapy for PAH patients.
